# The Role of Extracellular Vesicles in SARS-CoV-2-Induced Acute Kidney Injury: An Overview

**DOI:** 10.3390/life14020163

**Published:** 2024-01-23

**Authors:** Carter Bernal, Christiane How-Volkman, Madison Spencer, Ahmed El-Shamy, Ashraf M. Mohieldin

**Affiliations:** 1 College of Graduate Studies, California Northstate University, Elk Grove, CA 95757, USA; 2 College of Medicine, California Northstate University, Elk Grove, CA 95757, USA

**Keywords:** SARS-CoV-2, acute kidney injury, extracellular vesicles, COVID-19

## Abstract

The severe acute respiratory syndrome coronavirus 2 (SARS-CoV-2) has affected millions worldwide since its outbreak in the winter of 2019. While extensive research has primarily focused on the deleterious respiratory effects of SARS-CoV-2 in recent years, its pan-tropism has become evident. Among the vital organs susceptible to SARS-CoV-2 infection is the kidney. Post SARS-CoV-2 infection, patients have developed coronavirus disease 19 (COVID-19), with reported incidences of COVID-19 patients developing acute kidney injury (AKI). Given COVID-19’s multisystemic manifestation, our review focuses on the impact of SARS-CoV-2 infection within the renal system with an emphasis on the current hypotheses regarding the role of extracellular vesicles (EVs) in SARS-CoV-2 pathogenesis. Emerging studies have shown that SARS-CoV-2 can directly infect the kidney, whereas EVs are involved in the spreading of SARS-CoV-2 particles to other neighboring cells. Once the viral particles are within the kidney system, many proinflammatory signaling pathways are shown to be activated, resulting in AKI. Hence, clinical investigation of urinary proinflammatory components and total urinary extracellular vesicles (uEVs) with viral particles have been used to assess the severity of AKI in patients with COVID-19. Remarkedly, new emerging studies have shown the potential of mesenchymal stem cell-derived EVs (MSC-EVs) and ACE2-containing EVs as a hopeful therapeutic tool to inhibit SARS-CoV-2 RNA replication and block viral entry, respectively. Overall, understanding EVs’ physiological role is crucial and hopefully will rejuvenate our therapeutic approach towards COVID-19 patients with AKI.

## 1. Introduction

The global outbreak of novel pneumonia cases of unknown cause was first seen in Wuhan, China. Now, the pathogenic viral source of these cases is better known as severe acute respiratory syndrome coronavirus 2 (SARS-CoV-2) [1]. The family of coronaviruses has been ever-present, and infection with SARS-CoV-2 marks the third epidemic in the 21st century [2,3]. In fact, the genomic characterization of SARS-CoV-2 demonstrated an 88% resemblance to the bat-derived severe acute respiratory syndrome (SARS)-like coronaviruses, a 79% resemblance to the severe acute respiratory syndrome (SARS-CoV), and a 50% resemblance to the Middle East respiratory syndrome coronavirus (MERS-CoV) [4]. Despite its high mortality rate, SARS-CoV-2 causes a wide range of clinical presentations, including but not limited to psychiatric disorders, neurological disorders, endocrine disorders, respiratory disorders, and kidney disorders [5,6,7,8,9,10,11,12,13,14,15]. It has also been reported that there is a high risk of developing renal disorders, even in patients with mild COVID-19 [16,17]. Mechanistically, SARS-CoV-2 has been shown to invade and damage kidney tissue directly via various proinflammatory factors, inducing acute kidney injury (AKI) [13,14]. Once the kidney has been infiltrated, the distribution of viral particles can be propagated by extracellular vesicles (EVs) [18,19]. However, the cause-and-effect relationship between SARS-CoV-2, AKI, and EVs remains largely unexplained. This review manuscript will focus on discussing the pathogenesis of SARS-CoV-2-induced AKI, the various roles of EVs in SARS-CoV-2-induced AKI, and potential future directions.

### 1.1. Transmission and Infection of SARS-CoV-2 into Host Cells

**Figure 1:**
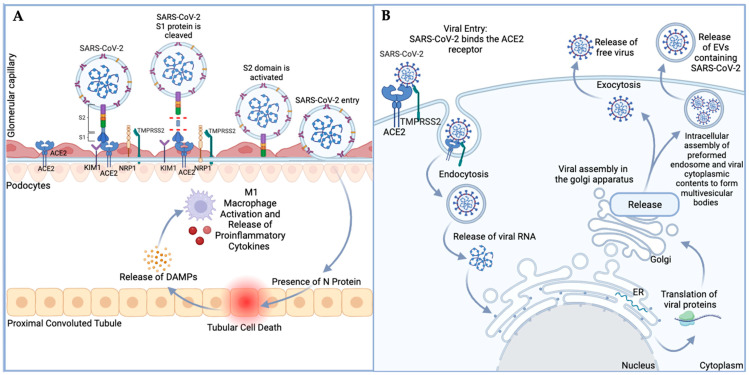
Proposed mechanism of SARS-CoV-2 entry and release of EVs containing viral cargo in kidney. (**A**). ACE2 expression on podocyte cell surface facilitates SARS-CoV-2 entry into Bowman’s capsule. Viral entry results in downregulation of ACE2 expression and renal tissue damage, which further stimulates macrophage activation and inflammatory cytokine release leading to a local inflammatory response. (**B**). SARS-CoV-2 endocytosis leads to viral uncoating and release of viral RNA, which is translated to viral proteins. Viral components are trafficked to the Golgi apparatus and subsequently released via exocytosis of either free virus or packaged into EVs. DAMPs, danger-associated molecular patterns; ER, endoplasmic reticulum. (Figure created with Biorender.com; accessed on 2 January 2024).

SARS-CoV-2 is a positive-sense single-stranded RNA virus transmitted via aerosols and respiratory droplets that infect the upper respiratory tract via the angiotensin converting enzyme (ACE2) receptor [20,21,22]. Successful transmission between those infected and their surrounding peers has been thought to be correlated with ACE2 receptor binding and docking [23,24]. SARS-CoV-2 is classified as a beta-coronavirus, and its structure includes a spike glycoprotein that is 1274 amino acids long, consisting of the S1 and S2 regions, which mediate cellular attachment [4]. The SARS-CoV-2 receptor binding domain (RBD) is harbored within the S1 region of the spike glycoprotein, while the S2 domain assists in membrane fusion [25,26]. Upon binding of the S1 region of the spike glycoprotein to the ACE2 receptor, the host protease, transmembrane protease serine 2 (TMPRSS2), proteolytically cleaves the spike glycoprotein into two segments at the S1/2 site and the S2′ site. This cleavage plays an integral role in priming the SARS-CoV-2 spike protein, facilitating the fusion and overall entry process of SARS-CoV-2 [26,27,28,29]. Although SARS-CoV-2 has been shown to have a multiorgan tropism, which includes the kidneys, the molecular mechanism by which SARS-CoV-2 enters the kidneys is complex and poorly understood [30,31,32,33]. For example, despite ACE2 and TMPRSS2 expression in renal tubules, some studies implicate that SARS-CoV-2 infects kidney cells through neuropilin-1 (NRP-1) binding furin-cleaved substrates and kidney injury molecule-1 (KIM-1) via immunoglobulin variable Ig-like (Ig V) domain [34,35,36]. KIM-1 is a membrane protein present in the proximal tubule of the kidney, which aids in the internalization of viruses, such as hepatitis A, and is upregulated following ischemic kidney damage [37,38]. Notably, cells that express KIM-1 in the absence of ACE2 are susceptible to SARS-CoV-2 infection through endocytosis of nanoparticles (virosomes) that contain the spike protein (Figure 1A) [35]. However, once the host cell is infected with SARS-CoV-2, it has been reported that fragile double-membrane vesicles containing viral replication complex are assembled [39]. Further, these vesicles are released into the extracellular space (Figure 1B) [40]. Consequently, these studies suggest the potential role of EVs in the viremic phase of SARS-CoV-2, which may promote infection, transmission, and intercellular spread.

### 1.2. Extracellular Vesicles and Cell-to-Cell Communication

EVs are membrane-bound particles of intracellular origin that protrude from the cell membrane and play a part in the pathological and physiological condition of host cells [41]. In addition to their homeostatic role, EVs are released during cellular stress, activation, and apoptosis [42]. Many forms of EVs released from eukaryotic cells are used for intercellular communication [42,43]. Within EVs cargo, there is an abundance of biomolecules that have the ability to target and reprogram the morphology and function of recipient cells [41,44]. Early hypotheses suggest that the function of EVs was to dispose of unwanted intracellular compounds; however, through the evolution of research on cancer and infectious disease, it has been documented that EVs aid in the progression of many pathological diseases [45]. Moreover, EVs have since been used to analyze the exchange of biomolecules between cells [43].

Numerous viruses, including Epstein–Barr virus (EBV) [46], Kaposi’s sarcoma-associated herpesvirus (KSHV) [47], cytomegalovirus (CMV) [48], and hepatitis C virus (HCV) [49] are suspected of exploiting EVs to confer their infectivity. More specifically, in HIV-1 infections, EVs are documented to transfer viruses and viral components from infected macrophages to neighboring cells; thus, facilitating viral infection [50].

### 1.3. Extracellular Vesicles and Their Role in SARS-CoV-2 Pathogenesis

Currently, the use of EVs as a mechanism of viral spread in SARS-CoV-2 is not fully understood, but evidence has surfaced suggesting the role of EVs in viral propagation. Sun et al. recently analyzed sputum samples by transmission electron microscopy obtained from COVID-19 patients and detected EV-like vesicles in the vicinity of the SARS-CoV-2 virion [19]. This study included a simultaneous in vitro investigation of the EVs’ cargo isolated from SARS-CoV-2 infected VeroE6 cells, which are kidney epithelial cells of an African green monkey. Following SARS-CoV-2 infection, the isolated EVs from the VeroE6 cells revealed the presence of the SARS-CoV-2 nucleocapsid (N) and spike (S) protein. Moreover, healthy control VeroE6 cells exhibited a death characteristic of virus-infected cells 48 h after being subjected to EVs isolated from the SARS-CoV-2 infected VeroE6 cells, suggesting that the kidney-derived EVs are capable of transmitting intercellular viral infection in vitro [19]. Furthermore, in a study by Kongsomros et al., an in vitro model using human lung epithelial cells, Calu-3, investigated the composition of EVs released from SARS-CoV-2 infected cells. Isolated EVs from SARS-CoV-2 infected Calu-3 cells were found to contain the SARS-CoV-2 nucleoprotein, while EVs from the control were absent of viral components. These results demonstrate that EVs derived from human lung epithelial cells are capable of housing SARS-CoV-2 viral cargo in vitro, suggesting a potential EV-facilitated mechanism of SARS-CoV-2 infection [51].

Notably, the ACE2 receptor has been found to be expressed in EVs released by endothelial cells, providing evidence of ACE2 transfer from cell to cell [52]. In general, EVs play a role in facilitating viral infection via their ability to exchange mRNA and microRNA (miRNA) between host cells [53,54]. The mobile nature of protein expression, including the ACE2 receptor, encapsulated and within the membranes of EVs, further suggests that SARS-CoV-2 may utilize EVs to confer intercellular infectivity within the host. In addition, the identified SARS-CoV-2 RNA in plasma-derived EV cargo supported the evidence that SARS-CoV-2 may also make use of the host intercellular communication system to infect neighboring and distant cells [55]. Although plasma RNA levels of SARS-CoV-2 are present in the blood, these findings convey a plausible scenario in which host EVs serve as an additional mechanism to facilitate viral spread from the lungs to cell types with a tropism for EVs, such as the renal system [56,57]. Consequently, the detection of miRNAs delivered by EVs can be used to monitor the presence of SARS-CoV-2 infection. A recent study has shown that ACE2 and dipeptidyl peptidase 4 (DPP4) enzymes were found to be genomic biomarkers elevated in naso-oropharyngeal swabs of COVID-19 patients [58]. Further, Latini et al. demonstrated that increased levels of ACE2 and DPP4 were associated with low levels of the miRNA hsa-let7b-5p in naso-oropharyngeal swabs of COVID-19 patients [59]. The inverse relationship between the miRNA hsa-let7b-5p and the presence of the enzymes ACE2 and DPP4 reveals the utility of monitoring miRNA levels to track the presence of SARS-CoV-2 infection. Another study has also shown that a decrease in postsurgical miR-125a-5p levels was present in patients who developed AKI, and the relative decrease was proportional to the severity of AKI [60]. The documented instances of miRNAs serving as biomarkers in early disease detection and progression is a significant finding that calls for further investigation, as it may serve a critical role in detecting and monitoring SARS-CoV-2 induced AKI.

### 1.4. Extracellular Vesicles as an Indicator of Kidney Disease and COVID-19 Disease Outcome

Injury to the kidney and declining kidney function have historically been measured with blood urea nitrogen levels, creatinine levels, and proteinuria. With the lack of evolving tests, EVs and their contents present an exciting option to measure kidney function, as sampling urinary EVs can be a non-invasive disease biomarker. For example, sampling of urine Aquaporin-2 (AQP-2), an integral water channel, has been previously utilized as a marker for water balance disorders, such as diabetes insipidus [61,62]. Similarly, Pisitkun et al. demonstrated that EVs obtained from a patient’s urine can be used to detect and monitor disease states. The isolation of urinary extracellular vesicles (uEVs) followed by the use of liquid chromatography-tandem mass spectrometry for proteomic analysis revealed nearly 300 proteins within the uEVs, including multiple protein products associated with renal and systemic diseases [62]. Another crucial study has revealed the clinical differences in uEVs and cytokines, further emphasizing the fact that EVs can be used to determine disease severity [63]. Particularly, in this study the urine samples were collected from patients with COVID-19 disease during the first days of hospitalization. The analyses of these urine samples revealed a significantly higher level of EVs present in the urine when compared to the healthy controls. Additionally, urine samples from the severe to critical COVID-19 patients demonstrated a higher level of uEVs and immune mediators compared to the mild to moderate COVID-19 patients. Increased levels of uEVs were found to be significantly associated with urinary proinflammatory cytokines such as TNFα, IL-1α, IL-1β, IL-16, and IL-17A. It has also been reported that uEVs may play a role in facilitating intra-nephron communication between the glomerular and tubular regions [64,65]. In a rodent-animal model study, AKI was shown to increase the renal biodistribution of intravenously injected labeled EVs. In addition, this study demonstrated that the characteristics and functional nature of miRNAs contained in uEVs are capable of identifying the stage and progression of AKI. For example, the elevation of certain miRNAs, including miR-16, miR-24, and miR-200c, were detected in the urine during AKI and associated with altered mRNA expression [66]. These findings further support the evidence that uEVs may serve as an early detection point to assess a patient’s clinical course and outcomes.

Another study has analyzed serum samples from 31 patients with mild COVID-19 symptoms at the time of their hospital admission to identify EV biomarkers capable of serving as a predictive marker for the severity of COVID-19. Of the 31 patients analyzed, 9 developed severe COVID-19 symptoms, and 22 patients progressed with mild COVID-19 symptoms. It was determined that COPB2 protein, a subunit of the Golgi coat complex subunit Beta 2 found inside EVs, served as a predictive biomarker for COVID-19 disease severity. High levels of COPB2 within EVs upon hospital admission were associated with patients who progressed with mild COVID-19 symptoms in comparison to those with severe COVID-19 disease outcomes and healthy controls [67].

Furthermore, the circulating EVs in plasma of patients diagnosed with COVID-19 were analyzed and demonstrated differential expression, suggesting EVs utility in predicting disease progression and outcome. The proteomic analysis of plasma EVs obtained from COVID-19 patients demonstrated the presence of molecules that facilitate COVID-19-associated tissue damage and organ dysfunction, including those involved in the immune response, the inflammatory response, the coagulation pathway, and the complement pathway. Between the cohorts, 157 EV proteins in the critical COVID-19 cohort and 97 EV proteins in the non-critical cohort were significantly regulated, suggesting the existence of several potential EV-associated biomarkers that correlate with the COVID-19 disease severity. For example, in critical COVID-19 patients, the level of EVs enriched proteins (CRP, A1AG1, A1AG2, CXCL7, SAMP, and ZA2G) demonstrated a positive correlation with disease severity. Differential enrichment of such proteins suggests the potential to utilize EVs as a diagnostic tool to anticipate disease progression, outcome, and patient response to therapy [55].

## 2. Acute Kidney Injury (AKI), EVs, and COVID-19

Despite Kidney Disease Improving Global Outcomes (KDIGO) current efforts to update the clinical practice guidelines for AKI and acute kidney disease (AKD), AKD is presently defined as an alteration of kidney structure and/or function for less than a 3-month period. Furthermore, AKI is classified as a subset of AKD, specifically referring to the emergence of symptoms within 7 days as a result of numerous etiologies, one of which being COVID-19. AKI is staged for severity based on the criteria of serum creatinine (SCr) and urine output [68]. The staging criteria for AKI can be found in Table 1. The etiology of AKI is often categorized as either pre-renal, intrinsic, or post-renal. Pre-renal causes include any extra-renal disease that may cause hypoperfusion to the renal parenchyma, such as sepsis, shock, and heart failure. Intrinsic AKI describes true renal disease, whereas post-renal etiologies of AKI include any obstruction or blockage of urine flow.

As SARS-CoV-2 began to spread globally, reports regarding the relationship between AKI and COVID-19 were noticeable, and incident rates were as low as 0.5% in China [69] and as high as 80% in critically ill COVID-19 patients in France [70]. As time progressed and SARS-CoV-2 infection spread to diverse geographic regions around the world, large cohort studies from Africa [71], China [72], England [73], India [74], Iran [75], Italy [76], Poland [77], Portugal [78], Spain [79], UK [80,81,82], and USA [83,84] have reported varying incidence rates of AKI among patients with COVID-19. The outcomes of these studies are summarized in Table 2.

Although numerous cohort studies spanning the globe report varying AKI incidences among COVID-19 patients, there exists a gap in the literature explaining the exact etiology of this disparity. Among COVID-19 patients worldwide, this variance is likely explained by different demographics, hospital admission threshold, in-hospital care, genetic predisposition, variance in AKI diagnosis criteria, temporal differences in kidney function assessment, and availability of treatment. However, there was little variance in study design, inclusion, and exclusion criteria that was notable between clinical studies discussed in Table 2. All studies were found to have used KDIGO AKI staging criteria, as depicted in Table 1, and verified the presence of SARS-CoV-2 infection in patients through diagnostic testing.

Despite the varying rates of incidence, AKI is a common clinical manifestation seen in COVID-19 patients and is associated with focal epithelial necrosis, glomerulosclerosis, and autolysis of renal tubular cells causing acute tubular necrosis (ATN) [85,86,87,88]. COVID-19-related AKI is presumed to be multifactorial; it is hypothesized to involve local and systemic inflammatory and immune responses, endothelial injury, and activation of coagulation pathways and the renin–angiotensin system, but the exact mechanism has yet to be elucidated [85,88].

In the early investigation of SARS-CoV-2, there was varying evidence on whether or not SARS-CoV-2 infected the kidney [89,90]. Emerging evidence has found that SARS-CoV-2 directly infects the kidney [91]. Radovic et al. discovered positive staining of S1 and NSP8 proteins in kidney parenchyma from COVID-19 patients, demonstrating evidence of active viral replication within the kidney [92]. These findings suggest that the AKI experienced by COVID-19 patients may result from direct infection of renal cells by SARS-CoV-2. Furthermore, infection and replication of SARS-CoV-2 in the renal parenchymal cells of COVID-19 patients could explain AKI presenting with manifestations of ATN and glomerulosclerosis. In addition, several studies revealed that COVID-19 patients showed increased levels of proinflammatory cytokines, including IL-1β, IL-2, IL-6, IL-10, IFN-γ, TNF-α, IFN-γ-inducible protein 10 (IP-10), granulocyte macrophage-colony stimulating factor (GM-CSF), and monocyte chemoattractant protein-1 (MCP-1) [93,94,95]. Further, it has been shown that the urine of COVID-19 patients contains increased levels of proinflammatory cytokines like IL-6, IL-8, and CXCL-10, and high urinary IFN-γ upon hospital admission proved to be a positive predictor of AKI in COVID-19 patients [96,97,98]. Moreover, a proteomic-based study demonstrated that changes in urinary cytokines are indeed associated with AKI development [99]. Thus, urinary proinflammatory cytokines such as TNFα, IL-1α, IL-1β, IL-16, and IL-17A and total uEVs in COVID-19 patients may identify patients that are prone to renal dysfunction [63]. Interestingly, many of these cytokines, such as MCP-1, IL-1β, and TNF-α, do overlap with the inflammatory mediators, which are upregulated by lipopolysaccharide (LPS)-induced AKI, demonstrating parallels between SARS-CoV-2-induced AKI and sepsis-induced AKI [99,100]. In addition, a comparative cohort study analyzing post-mortem autopsies on patients deceased of sepsis-induced AKI, SARS-CoV-2-induced AKI, and non-septic AKI controls found that glomerulitis and peritubular capillaritis seen in both sepsis-induced AKI and SARS-CoV-2-induced AKI were absent in non-septic AKI controls [101]. Further, the proteomic analysis by the latter study showed that the SARS-CoV-2-induced AKI overwhelmingly shared similar protein enrichment to sepsis-induced AKI, and only about 2% of the compared proteins were uniquely enriched between the two groups. Notably, the ceramide signaling pathway, which is a key factor in EVs biogenesis [102,103], was uniquely upregulated in SARS-CoV-2-induced AKI when compared with sepsis-induced AKI. However, there is no clear evidence that SARS-CoV-2 increases the likelihood of developing sepsis or vice versa [104], and more studies are warranted to establish such relationship.

The interaction loop between macrophages and EVs has also been shown to result in tissue injury commonly seen in AKI [105]. This interaction has been documented in multiple studies utilizing kidney tubular epithelial cells (TECs) and macrophages, modeling the renal landscape in which tubulointerstitial inflammation occurs. For example, in a study by Lv et al., purified TEC EVs rich in miRNA-19b-3p from an in vitro LPS-induced AKI model were applied to macrophages, which resulted in the upregulation of proteins such as p65, P-p65, and inflammatory cytokines such as MCP-1, IL-1β, IL-6, and TNF-α [106]. This administration of EVs displayed increased mRNA and protein levels of an M1 macrophage marker, iNOS, suggesting that TEC EVs polarize macrophages to their pro-inflammatory subtype. Furthermore, Li et al. collected EVs containing miRNA-23a derived from TECs subjected to hypoxic conditions. These EVs were found to activate macrophages and were then subsequently investigated in an in vivo model to determine their efficacy in reproducing renal tissue injury. One day after the injection of the EVs into murine renal parenchyma, there was the presence of increased inflammatory cells in the tubular interstitium and increased mRNA expression of the similarly reported upregulated inflammatory factors MCP-1, IL-1β, and TNF-α [107]. These supportive studies suggest the crosstalk between kidney TECs, EVs, and macrophages may alter inflammatory responses and induce kidney damage by polarizing macrophages to their M1 subtype. Notably, the overexpression of the SARS-CoV-2 N protein in the diabetic kidney of db/db mice has been shown to increase the infiltration of M1 proinflammatory macrophages via a macrophage-inducible C-type lectin (Mincle) pathway [108]. In addition, more studies have demonstrated the presence of SARS-CoV-2 N protein accumulations in the kidney tubular epithelium of patients with COVID-19 [91,109,110,111]. Another immunoelectron microscopy study revealed the accumulations of SARS-CoV-2 N protein in one patient previously diagnosed with COVID-19 three months prior to the manifestation of kidney failure [112]. Thus, the urinary SARS-CoV-2 N protein may serve as an indicator of the likelihood of AKI development, which may provide clinicians with insights into the severity of COVID-19 [113].

## 3. Extracellular Vesicles as a Treatment Option for COVID-19

**Figure 2:**
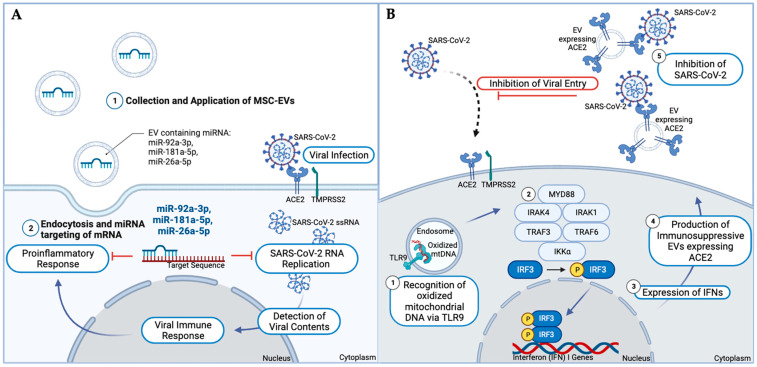
Proposed mechanisms of EVs as treatment options for COVID-19 Infection. (**A**). Application of EVs derived from MSCs has been shown to carry miRNA that complementarily binds to key target sequences, which inhibit SARS-CoV-2 RNA replication and the proinflammatory response that occurs with SARS-CoV-2 viral infection. (**B**). TLR9 typically acts to detect CpG islands of viral and bacterial DNA inducing the downstream MYD88 pathway. It has been proposed that TLR9 may be activated by oxidized mitochondrial DNA from damage occurring during respiratory tract infections. In the above mechanism, there is no direct activation of DNA sensors by SARS-CoV-2, but oxidized mitochondrial DNA causes subsequent activation of TLR9, resulting in the expression of IFN I genes. Expression of IFN I influences the expression of immunosuppressive EVs that express the ACE2 receptor. These EVs expressing the ACE2 receptor, termed defensosomes, act as decoys to inhibit SARS-CoV-2. (Figure created with Biorender.com; accessed on 2 January 2024).

EVs possess various attributes that have facilitated investigation for their utilization in therapeutics. A particularly relevant function of EVs is their ability to exchange mRNA and miRNA between host cells [53,54]. In the setting of SARS-CoV-2 infection, the utilization of mesenchymal stem cell-dervied EVs (MSC-EVs) containing miRNA as a potential therapeutic is a developing area of interest. The immunotherapeutic mechanism of MSC-EVs involves miRNA binding to viral mRNA in a complementary fashion, ultimately destabilizing the mRNA and silencing translation. Highly expressed MSC-EVs miRNAs, including miR-92a-3p, miR-103a-3p, miR-181a-5p, miR-26a-5p, and miR-23a-3p, demonstrate the capacity to inhibit SARS-CoV-2 RNA replication and promote the suppression of host cell proinflammatory responses induced by viral infection (Figure 2A) [54,114]. In a randomized controlled clinical trial performed by Zarrabi et al., patients with a progressive phase of acute respiratory distress syndrome were divided into three groups: one receiving an intravenous therapeutic dose of MSCs, one receiving an intravenous therapeutic dose of MSCs followed by inhalation of MSC-EVs, and one group serving as the control [115]. Patients who received therapeutic MSCs with and without MSC-EVs inhalation had a significant reduction in inflammatory markers, but the additional administration of inhaled MSC-EVs showed a greater reduction in the level of inflammatory markers. Furthermore, Vaka et al. demonstrated that the presence of IL-1β, IL-2, IL-8, IL-10, and TNF-α, key proinflammatory cytokines commonly seen in COVID-19 patients with acute respiratory distress syndrome, did not impact the viability or paracrine production of bone marrow mesenchymal stem cells, heart-derived cells, and umbilical cord mesenchymal derived stem cells [115]. These findings demonstrate that MSC-EVs are not affected by exposure to the hostile proinflammatory environment they are intended to suppress.

In a study by Park et al., placenta mesenchymal stem cells (pMSCs) demonstrated ACE2 receptor gene expression similar to the lung, and the EVs isolated from such placenta mesenchymal stem cells (pMSC-EVs) showed a high expression of ACE2 mRNA [114]. It has been proposed that pMSC-EVs expressing the ACE2 receptor could potentially act to competitively inhibit SARS-CoV-2 infiltration. The potential antiviral effects of EVs expressing ACE2 have been further supported by Ching et al. after an investigation of the effects of SARS-CoV-2 on the respiratory system. In vivo, EVs expressing ACE2 receptors (ACE2+EVs) were isolated from bronchoalveolar lavage fluid (BALF) in critically ill COVID-19 patients. These patients were admitted to the intensive care unit (ICU) due to respiratory failure requiring invasive mechanical ventilation. Among the ICU patients, EVs were discovered to vary in the magnitude of ACE2 expression per EV and the quantity of ACE2+EVs isolated. Ching et al. suggested common comorbidities, including diabetes, may correlate to the reduction in the total amount of ACE2+EVs isolated but did not correlate to the magnitude of ACE2 expression per EV. Despite the EVs’ variation among patients, the presence of ACE2+EVs isolated in patients’ BALF correlated to a reduced length of stay in the ICU and required days of ventilation. Furthermore, an in vitro arm of this study investigated the role of defensosomes in SARS-CoV-2 infection. Deferensosomes are a small subset of EVs understood to be involved in host defense against bacterial infection. Defensosomes containing ACE2 receptors were found to serve as decoys that inhibit SARS-CoV-2 infection. Moreover, defensosomes were found to neutralize SARS-CoV-2 infection through the binding of ACE2 present on defensosomes to the S protein on the SARS-CoV-2 virion. The defensosome-virion binding reveals that ACE2-expressing EVs are capable of binding and clustering SARS-CoV-2 virions, inhibiting spike protein fusion with host cells. The suspected mechanism through which SARS-CoV-2 can promote defensosomes is via the generation of oxidized mitochondrial DNA (mtDNA). Although toll-like receptor 9 (TLR9) commonly binds viral and bacterial DNA, it is hypothesized that oxidized mtDNA binds TLR9, leading to the downstream generation of interferons (IFNs) via the MYD88 pathway. IFNs were found to elicit the production of immunosuppressive EVs that may neutralize viruses, competitively inhibiting SARS-CoV-2 viral entry. Following this determination, genomic analysis was performed on patients with ACE2+EVs isolated in BALF, revealing increased expression of genes that facilitate antiviral signaling, like IFNs, strengthening the association between antiviral signaling and the production of defensosomes (Figure 2B) [116].

## 4. Limitations and Future Directions

Currently, there is a lack of sufficient studies investigating the role of EVs in SARS-CoV-2-induced AKI. Further, the potential implementation of treatment approaches via EVs for COVID-19 are still in the early stages. The field of MSC-EV therapy is a developing area of interest, with emerging clinical trials recruiting patients [117], though there are currently no FDA-approved MSC-EV therapies available. This review also acknowledges certain limitations, including reliance on small cohort and post-mortem studies, which may pose challenges in making broad generalizations due to their constrained sample sizes.

For future directions, a comprehensive understanding of the interactions between EVs and SARS-CoV-2 in AKI will pave the way for innovative diagnostic tools and targeted therapies. Thus, more targeted clinical studies are needed to demonstrate the efficacy and safety of MSC-EV therapies, and to establish the concept of defensosomes in hindering SARS-CoV-2-induced AKI. The evolving landscape of EV research in SARS-CoV-2-induced AKI holds promise for advancing our knowledge and improving clinical outcomes for affected individuals.

## 5. Conclusions

EVs play a major role in propagating SARS-CoV-2 during the viremic phase. Many studies are now supporting the evidence that urinary EVs can be used to assess and monitor AKI in patients with COVID-19. In addition, more innovative and hopeful studies are suggesting that EVs can be clinically useful to slow or inhibit the transmission and intercellular spread of SARS-CoV-2. Overall, the involvement of EVs in SARS-CoV-2-induced AKI represents a novel avenue for understanding the complex interplay between the virus and renal tissues. Further research is needed to unravel the precise mechanisms and potential therapeutic applications of EVs in mitigating kidney injury associated with COVID-19.

## Figures and Tables

**Table 1:** AKI Staging Criteria Outline of stages 1–3 of AKI, including stage 3 in the specific cohort of patients under 18 years old. Serum creatinine (sCr), micromole (μmol), liter (L), milliliter (mL), kilogram (kg), hour (h), deciliter (dL), milligram (mg), minute (min).

Stage	Description
Stage 1	sCR 1.5–1.9 times baseline or ≥0.3 mg/dL (≥26.5 μmol/L) increase and/or urine output < 0.5 mL/kg/h for 6–12 h
Stage 2	sCR 2.0–2.9 times baseline and/or urine output < 0.5 mL/kg/h for ≥12 h
Stage 3	sCR 3.0 times baseline or increase in sCR to ≥4.0 mg/dL (≥353.6 μmol/L) or initiation of renal replacement therapy
Stage 3 for patients <18 years old	Decrease in eGFR to <35 mL/min per 1.73 m^2^ and/or urine output <0.3 mL/kg/h for ≥24 h or anuria for ≥12 h

**Table 2:** Reports of varying incidence rates of AKI among patients with COVID-19 around the world. Estimated glomerular filtration rate (eGFR), end-stage kidney disease (ESKD), serum creatinine (sCr), chronic kidney disease (CKD), renal replacement therapy (RRT), polymerase chain reaction (PCR).

Author (Year)	Country	Sample Size	Male Sex (%)	Mean/Median Age (Years)	AKI (%)	Exclusion Criteria of the Cohort Study	Method of SARS-CoV-2 Diagnostic Test
Hung et al. (2022) [71]	Africa	990	92.10%	68	392 (39.6%)	History of ESKD, baseline eGFR < 15 mL/min/1.73 m^2^, no sCr levels recorded	PCR testing of nasopharyngeal specimen
Chen et al. (2021) [72]	China	1851	48.00%	62	115 (6.7%)	Lack of renal function tests	PCR testing of nasal and pharyngeal specimens
Bell et al. (2021) [73]	England	448	54.80%	69.4	118 (26.3%)	ESKD, dialysis, kidney transplant, or no sCr levels recorded	Positive COVID-19 swab
Sindhu et al. (2022) [74]	India	2650	81.60%	62.6	190 (7.20%)	Stage 5 CKD on dialysis	PCR testing of nasopharyngeal specimen
Rahimzadeh et al. (2021) [75]	Iran	516	62.80%	57.6	194 (37.6%)	History of hemodialysis or ESKD	PCR testing of oropharyngeal, nasopharyngeal, or endotracheal specimens, or symptoms consistent with COVID-19
Scarpioni et al. (2021) [76]	Italy	1701	64.30%	72.8	233 (13.7%)	ESKD, kidney transplant, or lack of two consecutive sCr determinations	PCR testing of nasopharyngeal specimen
Kilis-Pstrusinska et al. (2021) [77]	Poland	1958	52.10%	62.3	237 (12.1%)	AKI at admission	PCR testing
Marques et al. (2021) [78]	Portugal	544	56.30%	66.2	339 (62.3%)	CKD on RRT, discharged or deceased < 1 week after hospital admission	PCR testing of nasopharyngeal specimen
Procaccini et al. (2021) [79]	Spain	3182	–	72	548 (17.2%)	Dialysis, CKD Stage 5, or on RRT	PCR testing, clinical suspicion based on epidemiological data, blood parameters, and imaging
Sullivan et al. (2021) [80]	UK	41,294	62.60%	68	13,000 (31.5%)	Long-term dialysis, nosocomial infection, or readmission to hospital	PCR testing or clinical suspicion
Jewell et al. (2021) [81]	UK	1248	58.80%	69	487 (39.0%)	Probable hospital-acquired COVID-19, ESRD requiring RRT, or kidney transplant	Positive nasopharyngeal specimen, symptoms consistent with COVID-19, and a first positive SARS-CoV-2 swab test on, or up to 7 days after admission
Wan et al. (2021) [82]	UK	1855	60.50%	65	455 (24.5%)	Lack of sCr data or history of ESKD	PCR testing
Strohbehn et al. (2021) [83]	USA	1091	49.50%	67	251 (23.0%)	No recent baseline sCr, eGFR < 15, or on dialysis	PCR testing and hospitalized within 2 weeks of first positive test
Hsu et al. (2022) [84]	USA	4221	63.50%	61	2361 (56.0%)	Lack of data, baseline sCr, or kidney function at discharge, on dialysis, or hospitalized at last follow-up	Laboratory-confirmed SARS-CoV-2
